# Predictors of current suicidal ideation in a multi-diagnostic sample of individuals with eating disorders

**DOI:** 10.1186/s40337-023-00789-w

**Published:** 2023-06-08

**Authors:** Alyssa M. Izquierdo, Jillian D. Nelson, Alyssa Daza, Alexandra Gasbarro, Rebecca Hardin, Joanna Marino, Sarah Fischer

**Affiliations:** 1 Department of Psychology, George Mason University, 4400 University Drive, Fairfax, VA 22030 USA; 2 Potomac Behavioral Solutions, Arlington, VA USA

**Keywords:** Suicidal ideation, Eating disorders, Disordered eating, Suicide attempts, Nonsuicidal self-injury

## Abstract

**Background:**

Individuals with eating disorders (EDs) have high rates of suicidal ideation (SI) and attempts (SA). Fasting, body dissatisfaction, binge eating and purging have been associated with SI in non-clinical samples, individuals with anorexia nervosa or low-weight EDs, and a multi-diagnostic sample. However, few studies have examined how ED symptoms contribute to risk for SI in conjunction with other well-established risk factors, such as nonsuicidal self-injury (NSSI) and past SA. The aim of this study was to examine which ED symptoms contribute unique risk for current SI in a multi-diagnostic, clinical sample when statistically adjusting for gender, NSSI, past SA, and past SI.

**Methods:**

We conducted a chart review of 166 individuals who presented for ED treatment at an outpatient facility and signed informed consent. Initial intake interviews were coded for the presence versus absence of fasting, fear of weight gain, binge eating, purging, excessive exercise, restriction, body checking, self-weighing, and body dissatisfaction, as well as NSSI, past SA, past SI, and current SI.

**Results:**

A total of 26.5% of the sample endorsed current SI. In a logistic regression analysis, identifying as male (*n* = 17) or having a non-binary gender identity (*n* = 1), the presence of fasting, and past SI were all significantly associated with increased odds of current SI, whereas excessive exercise significantly decreased odds of current SI. Fasting was equally common across all diagnostic groups.

**Conclusions:**

Future research should establish the temporal relationship between fasting and SI to better inform intervention.

## Abstract (plain language summary)

Individuals with eating disorders are at high risk for suicidal thoughts and attempts. This study used chart review data from individuals with a wide range of eating disorder diagnoses who completed an intake interview for treatment and signed informed consent for research. We examined predictors of suicidal ideation at the time of the intake. Our final sample for data analysis included 148 women, 17 men, and one non-binary individual. We found that identifying as a man or with a non-binary gender identity, having a history of previous suicidal thoughts, and fasting all increased the likelihood that a patient with an eating disorder had suicidal thoughts in the period of time around their intake interview. Excessive exercise behavior decreased the likelihood that a patient with an eating disorder had current suicidal thoughts. This study provides important information about suicide risk in eating disorders. Unlike most studies on this topic, the analysis included well-established risk factors for suicidal ideation. It may be helpful to understand more about why fasting increases risk for suicidal thoughts, even when accounting for other risk factors.

## Introduction

Individuals with eating disorders (EDs) have high rates of suicidal ideation (SI), suicide attempts (SA), and non-suicidal self-injury (NSSI; [1]). Lifetime NSSI, or the act of inflicting physical harm to one’s body without suicidal intent, occurs in 27.3% of individuals with EDs [2]. Suicidal behaviors, such as SI and SAs, are also highly prevalent in ED populations [3]. NSSI and SAs are more common among individuals with bulimia nervosa (BN) and anorexia nervosa (AN) binge/purge subtype (AN-BP) compared to those with AN restricting subtype (AN-R) [1, 4, 5]. And, one of the most common causes of death in AN is suicide [6, 7].

Historically, the focus of research on suicidal behavior in EDs has centered on diagnostic group differences in these behaviors, and factors within diagnostic groups that are linked to ideation and attempts [8]. Given that individuals with almost all types of ED diagnoses are at high risk for suicide, it may be informative to examine the impact of symptoms, rather than diagnostic categories, on SI. A growing body of literature has examined the impact of specific symptoms of EDs themselves, rather than ED diagnosis, on vulnerability to self-injurious behaviors, including suicidality. Although NSSI does not involve intent to die, it is a robust predictor of SI and attempts [9]. Thus, this type of self-injurious behavior is often studied in conjunction with suicidal behaviors. Research has primarily focused on the impact of body dissatisfaction, binge eating and purging, and restricting and/or fasting on self-injurious behaviors. All of these ED symptoms have been correlated with suicidal behaviors, and several hypotheses regarding the function of this positive association have been proposed.

### Binge eating, purging, and SI

Binge eating and purging are positively associated with NSSI, SI, and SAs [4, 8, 10, 11]. There is already a large body of research linking binge eating and purging to emotion regulation (e.g., [12, 13]). Further, one of the most common functions of NSSI and suicidal behavior is to regulate negative emotion [14–16]. Several studies indicate that individuals with BN may engage in NSSI and SAs to alleviate negative affect [17–19]. Deficits in emotion regulation involve heightened emotional responses, sensitivity to emotionally salient stimuli, and difficulty regulating these responses [20]. Within this framework, binge eating, purging, and self-injurious behaviors serve as dysfunctional attempts to regulate emotions. Researchers also suggest that purging may function similarly to suicidal behavior since it is provocative and harmful. In a study of patients with EDs who were undergoing treatment, purging predicted the intensity and presence/absence of SI at discharge, whereas binge eating and restriction did not [21].

### Fasting, restriction, and SI

Recent studies suggest that fasting may predict NSSI, SI, and SAs. For instance, fasting was uniquely related to SI in a study of adolescents with low-weight EDs [22]. Another study found that fasting was uniquely related to lifetime NSSI in a non-clinical sample [23], and restricting distinguished individuals with SI from those without SI in another large non-clinical sample [24]. Notably, these studies did not include past self-injurious behaviors as predictors in conjunction with ED symptoms in their analyses. Restriction or fasting may relate to suicidal or self-injurious behavior in ways similar to body dissatisfaction, bingeing, and purging. The emotion regulation theory of restriction posits that individuals with AN may restrict as a way to avoid or reduce negative affect [25]. Thus, restriction may be linked with suicidal or self-injurious behavior in EDs because both behaviors may serve to downregulate aversive emotional experiences. In addition, deficits in interoceptive awareness may link ED symptoms and SI [1, 26]. Patients with EDs who reported current SI had higher levels of ED pathology, including dietary restraint, and impaired interoceptive awareness compared to patients without SI [26].

One notable aspect of studies on dietary restriction and its relation to suicidal behavior and NSSI is how restriction is defined. For instance, studies that found these associations defined restriction as fasting for 8 h or more [22, 23]. Although all forms of food restriction may be harmful in individuals with EDs, it is possible that the recurrent effects of starvation may represent as a form of serious self-injury and painful experiences that can increase the risk of suicide [27]. Thus, it is important to consider a range of restrictive eating behaviors, such as fasting vs. dieting or cutting out food groups, when examining their association with suicidal behavior and NSSI.

### Body dissatisfaction and SI

Body dissatisfaction is associated with increased risk of SI in individuals with EDs [28]. One prominent theory that helps explain this association is the interpersonal-psychological theory of suicide (ITPS), which posits that SI comes from people having feelings of perceived burdensomeness (i.e., self-hatred or the thought that you are a burden to others; [29]) and thwarted belongingness (i.e., unmet need of social connectedness; [8]). Body dissatisfaction may be linked to these concepts in several ways [28]. For example, many individuals with EDs may withdraw from social activities or perceive themselves to have low social support [28]. Low self-esteem may be connected to both body dissatisfaction and the perception that one has low value to others [28]. This effect has been shown longitudinally in large non-clinical adolescent samples [8, 30], and may be linked to distorted thoughts regarding burdensomeness and belongingness. For example, in a cross-sectional study of women with EDs, body dissatisfaction and fasting were shown to be indirectly related to SI via higher perceived burdensomeness [28]. Other researchers have found that greater ED pathology was associated with suicidal behavior via perceived burdensomeness and thwarted belongingness [31]. Although Forrest and colleagues controlled for depressive symptoms in their analyses, other predictors of SI, such as NSSI and past SAs, were not included in their study [28].

### Other ED behaviors and SI

Studies have shown that excessive exercise is related to suicide risk; however, research on the link between these two constructs is very limited [32, 33]. Researchers suggest that excessive exercise is a potentially painful behavior that may increase pain tolerance and the capability of suicide, which are components of the ITPS [32]. Driven exercise can also serve as an emotion regulation strategy [34]. There is a dearth of research on the relationship of other behaviors common in individuals with EDs, such as body checking and weighing oneself, to suicidal ideation. Both of these behaviors are linked to body dissatisfaction, trigger shame and negative affect, and increase critical thoughts about one’s appearance [35–37]. Thus, it is possible that these behaviors may be linked to SI in individuals with EDs via increases in acute negative affect or via increased burdensomeness.

### Current study

The aim of this study is to examine which ED symptoms contribute unique variance to current SI when including the effects of past SI, SAs, and NSSI in a mixed-diagnostic clinical sample of individuals with EDs. Notably, the proposed mechanisms for these associations (burdensomeness and belongingness, emotion regulation, impaired interoceptive awareness) may be common across all ED diagnoses. In the current study, we examined the association of multiple symptoms of EDs (body dissatisfaction, fasting, restriction, binge eating, purging, body checking, excessive exercise, weighing self, and fear of weight gain) to current SI. Previous studies that have explored this issue have used non-clinical samples, compared individuals with different ED diagnoses to each other, or used a homogenous sample of individuals with one diagnosis (e.g., [22] but see [28]). Thus, in the current study, we examined the association of ED symptoms to current SI in a multi-diagnostic, clinical sample of individuals with several types of ED diagnoses.

Previous studies have largely examined ED symptoms as risk factors or correlates for SI, without taking into account other established vulnerability factors. However, a large body of literature documents that NSSI, previous SAs, and past SI are also predictors of current SI [9, 38, 39]. Determining whether associations between SI and ED symptoms exist when these other factors are considered is understudied. Bodell and colleagues [40] examined predictors of SI in a sample of individuals with EDs, and included past SI, psychological impairment, and ED symptoms as predictors. However, their sample consisted only of individuals with AN [40]. Joiner and colleagues [21] also included past SI, in addition to purging, binge eating, and restriction, in a longitudinal study examining risk for SI in individuals with EDs. The current study will expand upon previous work [21, 40] by including ED symptoms and self-harming behaviors (NSSI, past attempts, and past SI) as additional predictors of SI.

As previous studies have documented associations between binge eating, purging, body dissatisfaction, and fasting to SI, we hypothesized that these symptoms would be positively associated with SI in this sample. We included several other ED behaviors and symptoms in addition to these. First, there is a dearth of research on the relationship of other ED behaviors and SI, such as body checking and excessive exercise. Second, it is possible that behaviors such as body checking or weighing oneself may be more proximally linked to SI as they increase negative affect and self-critical thoughts.

## Method

### Study design and analysis plan

The senior author and first three authors conducted a retrospective chart review (RCR) of clinical intake interview data collected across a five-year period in an outpatient facility specializing in the treatment of EDs. A RCR allows researchers to retrospectively access data that exists in the patient’s medical record for research purposes. Data from the structured clinical intake interview were entered into a database and each client was assigned a unique study ID number; personally identifiable health information was not included in the database. ED diagnosis was taken from the chart and entered in the database. Symptoms (i.e., ED symptoms, SI, NSSI, and SAs) were coded as dichotomous variables (present vs. absent). Prior to testing our main hypotheses, we conducted descriptive analyses. We utilized chi square analyses to examine potential differences in rates of NSSI, past SI, SAs, and current SI across diagnostic groups.

As all symptom variables were coded as present vs. absent, binary logistic regression analyses were conducted to test our hypotheses. The dichotomous variable representing the presence vs. absence of current SI was entered as the outcome variable. We entered gender, NSSI, past SI, and SAs, and the following ED symptoms: fasting, fear of weight gain, binge eating, purging, excessive exercise, restriction, body checking, weighing self, and body dissatisfaction as predictors. Finally, we conducted a follow up analysis. It is possible that a given ED symptom may occur more frequently in one diagnostic category vs another. If a particular ED symptom is significantly associated with current SI and occurs at equivalent rates across all diagnostic categories, this may be indicative of a transdiagnostic ED-related vulnerability factor for suicidal thoughts. However, if a given symptom occurs more frequently in one diagnostic group vs. another (such as AN-R or AN-BP), then this may be indicative of vulnerability being higher within a particular diagnosis. Thus, we conducted a chi square analysis of the presence vs. absence of significant ED symptom predictors across diagnostic groups.

### Participants

Participants were individuals who both presented for treatment for an ED at an outpatient treatment center between 2014 and 2018, and signed informed consent allowing chart data to be used for research. Every person who completed an intake interview was invited to sign informed consent for their chart data to be used for research purposes. Thus, there were no inclusion or exclusion criteria for the research. Inclusion criteria for the analysis presented here was that the individual presented for ED treatment. Individuals under the age of 18 years included in the sample had signed minor assent and had received parental consent for their information to be used for research. Over the chart review period, 228 clients presented for treatment for an ED, and 166 of them signed informed consent allowing chart data to be used for research. The data from those 166 individuals were used for this study. The study was approved by George Mason University’s institutional review board.

### Setting

Data were collected at a private practice outpatient treatment center that specializes in treatment for EDs, anxiety disorders, and borderline personality disorder in a large urban area outside of Washington, DC in the United States. The practice is a fee-for-service treatment center. As such, there is likely limited variability in the socio-economic status of the sample. Therapists have been trained in several empirically supported treatments, including Cognitive Behavior Therapy for eating disorders (CBT-E; [41]), Family Based Treatment (FBT) for AN [42], and Dialectical Behavior Therapy [43], among others. All clients who express interest in treatment at the practice are asked to complete a structured clinical intake interview that assesses a full range of psychiatric diagnoses, trauma history, developmental history, and suicide risk. Additionally, information is collected about previous treatment, treatment goals, and age of onset of symptoms. Following the intake interview, a preliminary assignment to treatment is made, based on this information. The preliminary diagnosis given during the intake interview is confirmed during the first two sessions by the assigned individual provider through additional follow-up structured clinical interviewing or self-report measures (based on the discretion of the assigned clinician) and placed in the chart.

### Measures

#### Intake interview

The primary measure used in the current study was the structured intake interview. The interview utilized the Structured Clinical Interview for DSM-IV: Clinician Version (SCID; [44]) questions for mood disorders, anxiety disorders, post-traumatic stress disorder, substance use disorder, EDs, and attention deficit hyperactivity disorder. As the practice specializes in outpatient treatment for EDs, additional questions were included regarding a wider range of ED behaviors than is available in the SCID (described below). Intake interviewers were trained in the use of the SCID and other intake questions. If the patient responded “yes” to a symptom question, then the interviewer asked several follow up questions. The interviewer asked for examples of the symptom or behavior, asked about the frequency of the behavior, and asked about the most recent behavior. If the responses were consistent with recognized definitions from the DSM or standard definitions in the field, then the interviewer coded the symptom as present. The presence vs. absence of a symptom was coded using a binary ‘yes/no’ variable. Additionally, the intake interview assessed length of illness, trauma history, treatment history, suicide risk, and assessment of history of NSSI and SI and attempts. The intake interviewers asked patients to self-identify their gender, and noted their responses in the chart. Individuals deemed at imminent risk for suicide during the intake interview were connected with emergency services.

#### Study variables

The following variables were used as predictor variables. Fasting was defined in the intake interview as skipping a meal and/or going for 8 h or more without food during the day (adapted from Fairburn and Beglin [46]). Body checking was defined as repeatedly checking one’s body for signs of weight gain, such as pinching flesh, feeling bones, trying on clothes, or examining body parts in the mirror [45]. Binge eating was defined as eating an objectively large amount of food in a short period of time with loss of control [44]. Purging was defined as self-induced vomiting, laxative use, or diuretic use [44]. Restriction was defined in the intake interview as following diet rules, cutting back on calories, or cutting out a food group [44]. Excessive exercise [46], body dissatisfaction [44], weighing self [44], and fear of weight gain [44] were also assessed. Past SAs were defined as harming oneself with the intent to die [41]. NSSI was defined as physically harming oneself without the intent to die [47]. Suicidal ideation was coded as ‘past’ if it had occurred prior to two weeks before the intake interview. Therefore, past SI could have occurred at any point in the patient’s lifetime, up until two weeks before the intake interview. SI was coded as ‘current’ if it was present during the intake interview, or during the two weeks leading up to and including the interview. Passive (e.g., wishing one was dead) or active (e.g., thinking about a specific plan to commit suicide) ideation [44] were collapsed for the purposes of this analysis. The outcome variable was the presence vs. absence of current SI.

## Results

### Sample descriptive statistics

Among the 166 participants, 89.1% identified as women, 10.3% identified as men, and the remaining individual self-identified as having a non-binary gender identity according to their responses in the intake interview. Participants had a wide range of ED diagnoses, with otherwise specified feeding and ED (OSFED) as the most common (48.2%), followed by AN-R (19.3%), and BN (15.1%). Other diagnoses included binge ED (BED; 9.0%), AN-BP (4.8%), and avoidant restrictive food intake disorder (ARFID; 3.6%). Age at intake ranged from 14 to 58 years, with a mean age of 26.54 (SD = 8.28). Average length of illness was 111.35 months (SD = 106.85 months), with a range of three months to 552 months. A total of 79.2% had at least one comorbid diagnosis. The number of comorbid diagnoses ranged from one to five, with a mean of 1.78. The modal number of comorbid diagnoses was 2 (31.1% of the sample).

### Missing data

A total of four participants did not have information in the intake data indicating whether or not they had current SI. An additional 36 participants did not have complete intake data regarding the presence vs. absence of either one or more ED symptoms (such as body checking) or NSSI, although all intake data indicated whether or not the participant binged, purged, engaged in excessive exercise, or endorsed some form of food restriction. Therefore, analyses were conducted for participants with complete data (final *N* = 126). An independent samples t-test indicated that there were no statistically significant differences in age at intake (t = 1.81, *p* < 0.07) and age at onset (t = 0.54, *p* < 0.60) for individuals who were included in the analysis (mean age at intake = 27.20 years; mean age at onset = 19.85 years) and those who were not (mean age at intake = 24.41 years; mean age at onset = 17.00 years). A chi square analysis of the binary gender variable indicated that there were no statistically significant gender differences in the sample that was included in the analysis and those who were not (*X*^2^ = 2.15, *p* < 0.12). No one in the excluded sample self-identified as having a non-binary gender identity. Finally, there were no differences in diagnostic categories between the two groups (*X*^2^ = 4.94, *p* < 0.42).

### Rates of NSSI, suicide attempts, and suicidal ideation

A total of 26.5% of the sample reported current SI at the time of intake. Among individuals who identified as men, 41.2% reported current SI, while 24.3% of individuals who identified as women reported current SI. A total of 48.8% of the sample reported a history of SI (47.2% of women and 58.8% of men). A total of 19.3% of the sample reported a past SA (18.9% of women and 17.6% of men), and 30.3% reported NSSI (30.6% of women and 23.5% of men). There were no statistically significant differences in the frequencies of current SI, past SI, NSSI, or SAs across diagnostic groups (Table 1).

**Table 1:** Frequencies and chi-square results for presence versus absence of NSSI and all suicide-related variables across diagnostic categories

	Current SI	Past SI	NSSI	Suicide attempt
*X*^2^ (5) (*p*)	6.47 (0.78)	7.15 (0.71)	9.34 (0.49)	10.35 (0.41)
*Diagnosis (n)*				
AN-R (32)	28.1%	43.8%	31.3%	9.4%
AN-BP (8)	37.5%	62.5%	50.0%	37.5%
BN (25)	16.0%	56.0%	29.2%	12.0%
BED (15)	26.7%	53.3%	26.7%	26.7%
ARFID (6)	50.0%	50.0%	0%	16.7%
OSFED (80)	26.3%	46.3%	31.3%	22.5%

AN-R = anorexia nervosa-restricting type; AN-BP = anorexia nervosa-binge/purge type; ARFID = avoidant/restrictive food intake disorder; BED = binge eating disorder; BN = bulimia nervosa; OSFED = other specified feeding or eating disorder; NSSI = nonsuicidal self-injury; SI = suicidal ideation

### Logistic regression analyses

One individual in the sample did not identify as a man or woman. Non-binary individuals are often excluded from analyses that include binary gender categories as variables. In this sample, the low number of non-binary individuals precluded group comparisons or the use of techniques such as effects coding. We did not want to place them in a gender group that was not representative of their identity. However, we did not wish to exclude this participant from the analysis, and thus have their data not represented at all. We placed the non-binary individual in the ‘men’ group, as men and individuals with marginalized gender identities have higher rates of completed suicide than women [48–50]. There is a large body of research on EDs in women. Therefore, we labeled the group with men and the non-binary participant as the reference group, framing this variable as ‘not identifying as a woman.’

The overall model was significant (Cox and Snell *R*^*2*^ = 0.23, and Nagelkerke *R*^*2*^ = 0.32) and correctly classified 73.6% of the sample (See Table 2). Three variables significantly increased the likelihood of current SI while statistically adjusting for all other variables. The first was gender; (OR = 4.88, 95% CI = 1.16 − 20.60; *p* < 0.03). Individuals who did not identify as a woman (17 men and 1 non-binary individual) had increased odds of current SI. The second was past SI; (OR = 4.69, 95% CI = 1.78 − 12.33; *p* < 0.002). The odds of endorsing current SI were 4.69 times higher if the individual also reported past SI. The odds of endorsing current SI were 3.23 times as high for those who endorsed fasting vs. those who did not; (OR = 3.23, 95% CI = 1.15 − 9.27; *p* < 0.03). Finally, results indicated that the presence of excessive exercise decreased the odds of endorsing current SI; (OR = 0.35, 95% CI = 0.13 − 0.98; *p* < 0.046) (Table 2).^1^

**Table 2:** Logistic regression of gender, ED pathology, NSSI, past suicidal ideation, and suicide attempts on the presence versus absence of current suicidal ideation in the complete sample

	*X*^2^ (df) (*p*)	Exp (B) (*p*)	95% CI
Overall Model	32.35 (13) (0.002)*		
Gender		4.88 (0.03)*	1.16–20.60
NSSI		0.47 (0.15)	0.17–1.31
Past SI		4.69 (0.002)*	1.78–12.33
Suicide attempt		0.46 (0.12)	0.17–1.26
Binge eating		0.29 (0.22)	0.22–1.56
Purging		0.98 (0.97)	0.35–2.76
Excessive exercise		0.35 (0.046)*	0.13–.98
Restriction		1.07 (0.36)	0.36–3.19
Body checking		2.41 (0.10)	0.85–6.82
Fasting		3.23 (0.03)*	1.15–9.27
Body dissatisfaction		1.29 (0.63)	0.46–3.62
Fear of weight gain		0.54 (0.23)	0.19–1.49
Weighing self		0.78 (0.28)	0.28–2.16

CI = confidence interval; ED = eating disorder; NSSI = nonsuicidal self-injury; SI = suicidal ideation

*p < 0.05

### Follow-up analyses

Given that fasting was associated with increased odds of current SI, and excessive exercise was associated with decreased odds of current SI, we conducted a chi square analysis to examine whether or not these symptoms occurred more frequently in any one diagnostic group. Results indicated that fasting was reported at equivalent rates across all diagnostic groups (*X*^2^(5) = 4.87,* p* < 0.43). Excessive exercise also occurred at equivalent rates across all diagnostic groups (*X*^2^ (5) = 8.66, *p* < 0.12).

## Discussion

The focus of this study is to examine the association of specific ED symptoms to current SI in a multi-diagnostic sample, when statistically adjusting for other risk factors such as NSSI and past SAs. Past studies have examined how ED symptoms impact risk for SI when including previous SI in statistical models [21, 40]. This study expanded upon this work by also including NSSI and past suicide attempts, both of which are robust predictors of SI and are common in individuals with EDs. We found that fasting was associated with increased odds of current SI, while other ED symptoms were not. This finding is consistent with a previous study that showed a unique association between fasting and SI in a sample of young women with AN [22]. Notably, there were no significant differences in rates of fasting, SAs, and current and past SI between diagnoses. This suggests that fasting is a potential transdiagnostic risk factor for SI. There are several possible mechanisms that may contribute to this relationship between fasting and SI. Fasting involves the most extreme restriction of food intake, which may function as a form of self-harm that can increase pain tolerance and capability of suicide [29]. Impaired interoceptive awareness may contribute to this link. Studies suggest that individuals with interoceptive deficits may feel disconnected from their bodies and thus be more prone to engage in self-harming behaviors such as NSSI or fasting [1]. Engaging in these behaviors can then increase pain tolerance, which has been proposed as a potential mediator of interoceptive deficits and SAs in women with EDs [51].

It is also important to consider the physiological effects of fasting and how this may impact risk for SI. Malnutrition impacts cognitive functioning, mood, and personality. It is well-established in the literature that decision making is impaired in EDs [52]. Importantly, impaired decision making is not detected in those recovered from AN, suggesting these deficits are related to the acute phase of illness [52]. Malnutrition in EDs caused by severe food restriction may uniquely increase risk for SI in this way.

Binge eating and purging behaviors, which were correlates of SI and other risk behaviors in other samples, were not significantly associated with increased odds of current SI when all other symptoms and risk factors were included in the model [1, 4, 5]. The equivalent rates of SI across diagnostic groups indicates that the relationship between fasting and SI was not limited to restrictive-type EDs. Binge eating and purging are often characterized as emotion regulation strategies [12, 13]. Although unhealthy, individuals who binge eat and purge perceive these behaviors as effective ways to alleviate acute negative affect. It is possible that these behaviors were not significantly associated with SI because they more effectively or quickly regulated negative emotion than behaviors such as fasting. However, this is speculative as we only have data on symptoms of EDs, not on constructs such as emotion regulation. Additionally, excessive exercise was associated with decreased odds of current SI in the complete sample, and occurred at equivalent rates across diagnostic groups. There is little research on the relationship of excessive exercise specifically to SI. Moderate exercise is linked to improved mental health, although these data are not specific to ED samples [53]. Both of these findings may be a function of this particular sample, and thus should be replicated in larger samples of treatment seeking individuals.

Identifying as a man or with a non-binary gender identity was associated with increased likelihood of current SI. Of note, this finding included a sample of 17 men and one non-binary individual. Almost half of the men in the study endorsed current SI, in contrast to 24.3% of the women. Research on the prevalence of SI and attempts in men with EDs is limited. One study found men with EDs had higher rates of past SAs than women [54], while another found men with AN endorsed lower rates than women [55]. Both studies had small sample sizes, so further research is needed. Men are less likely to ever seek treatment for an ED [56, 57]. Men who seek treatment do so at an older age and report longer illness duration [57]. Due to this, men may only engage with treatment when already experiencing high levels of distress or more severe psychopathology, including SI. This may be due to the stigma that men experience related to ED symptoms, as they have been viewed as “feminine” disorders [58]. Additionally, individuals with marginalized gender identities also have higher rates of SI and SAs than cis-gender individuals [49, 50]. Both men with ED symptoms and individuals with marginalized gender identities may experience thwarted belongingness due to the stigma they experience, and thwarted belongingness may increase risk for SI in these groups [59].

In addition, past SI increased the odds of current SI. The association between past and current SI has been well-established [9, 39, 60]. One limitation of the variable operationalizing past SI is that it was coded such that SI could have occurred at any point in the patient’s lifetime, including the period up to two weeks before the intake. Thus, someone who had SI a month prior to the intake, and also reported SI at the intake, would have been coded as having past SI and current SI. This may inflate the relationship between past and current SI. However, many more individuals reported past SI than current SI, and it was assessed throughout the lifespan. Therefore, it is likely that this positive finding was in fact consistent with previous literature on this topic.

Our study strengths include the inclusion of a diverse ED sample, and a comprehensive set of core ED symptoms and well-known risk behaviors related to SI. Limitations of the study include the cross-sectional design and potential sampling bias toward clients with higher levels of distress. One strategy for increasing generalizability is to recruit a community sample or clients who are not specifically seeking ED treatment. An additional limitation is the binary nature of the variables used in the analysis. Due to the structure of the intake interview, symptoms were coded in the chart as ‘present’ or ‘absent’. Thus, a continuous measure of symptoms was not available for use in the analysis. A continuous measure of symptoms would have provided a richer and more nuanced understanding of the relationship of these variables to each other. Related to this point, fasting was coded as present if the person deliberately went for eight hours or more without eating for weight loss or compensatory purposes, or if the person skipped a meal for weight loss or compensatory purposes. Individuals who skipped a meal may not have fasted as long as others, and so this behavior may not be as severe as other forms of fasting. The use of continuous measures of constructs such as binge eating, purging, and suicidal ideation may yield different results. For example, Joiner and colleagues [21] found that purging, measured with a multiple item scale, was associated with SI in a sample of individuals with EDs. An additional limitation is that we examined gender as a predictor using a binary category and had one participant with a non-binary identity. Because of these issues, the manner in which we examined gender as a predictor was limited.

As this was a cross-sectional study design, one cannot make inferences regarding the causality of the link between fasting and SI. Therefore, important future directions for research include the use of designs that elucidate the temporal nature of this relationship or examine how underlying variables may influence these relationships. Individuals with low mood who are suicidal may have low motivation to eat, and thus skip meals. Or, individuals who fast may have impaired cognition and increased dysphoria due to the malnourishment described above, which may lead to SI. Another future direction is to utilize a longitudinal design to elucidate how these predictors may have an effect on SI at treatment discharge. Finally, other mechanisms such as emotion regulation, self-punishment, or high pain tolerance may be a third variable that increases the likelihood of this association.

## Conclusions

In this treatment seeking multi-diagnostic sample, not identifying as a woman, having a history of SI and fasting were associated with increased odds of SI at the time of intake. Our findings underscore the importance of screening for fasting in individuals across all types of ED diagnoses when they present for treatment. Assessing factors such as emotion regulation, burdensomeness, and/or thwarted belongingness can identify underlying mechanisms of ED behaviors and self-harming behaviors that may increase an individual’s risk for SI. This information may help clinicians intervene in risk for suicide. If replicated, these findings may be helpful in enhancing suicide prevention and intervention efforts more broadly.

## Data Availability

The datasets generated and/or analysed during the current study are not publicly available because they are taken from patient medical records. The variables used in this study may be available in aggregate from the corresponding author on reasonable request.

## Abbreviations

AN: Anorexia nervosa
AN-BP: Anorexia nervosa, binge-purge subtype
AN-R: Anorexia nervosa, restricting subtype
BN: Bulimia nervosa
ED: Eating disorder
NSSI: Non-suicidal self-injury
RCR: Retrospective chart review
SA: Suicide attempts
SI: Suicidal ideation

## Footnotes

1 After running our analyses, we re-ran all our models with length of illness in the model. The findings remained consistent, with fasting, gender, and past suicidal ideation as significant predictors. A total of 33 participants were missing data from the intake interview on length of illness. Thus, we chose to exclude this variable in our final reported analyses as it would significantly reduce the sample size to include it, and the findings remained the same.
